# Plasma-Side Analysis of Chemical-to-Ion Flux Balance and Ion Energy-Angular Distributions in Ar/O_2_ Capacitively Coupled Plasmas for MoS_2_-Relevant Low-Damage Patterning

**DOI:** 10.3390/mi17080891

**Published:** 2026-07-25

**Authors:** Cheol Woong Kim, Geonwoo Park, Hae June Lee

**Affiliations:** 1 Department of Electrical Engineering, Pusan National University, Busan 46241, Republic of Korea; innerbubble@naver.com; 2 Memory Equipment and Component Technology Team, Samsung Electronics Company, Ltd., Hwaseong 18448, Republic of Korea; gw0.park@samsung.com

**Keywords:** low-damage plasma processing, Ar/O_2_ capacitively coupled plasma, MoS_2_ patterning, ion-to-radical flux ratio

## Abstract

Low-damage plasma processing of atomically thin $MoS_{2}$ requires simultaneous control of ion species, energy, and incident angle, yet the discharge mechanisms governing $Ar/O_{2}$ plasma and their connection to surface damage remain insufficiently understood. Here, we investigate the effect of the $Ar/O_{2}$ mixing ratio on the spatial distributions of charged particles, the plasma potential, and the substrate-incident ion energy and angular distributions in a low-voltage, single-frequency capacitively coupled plasma using a two-dimensional particle-in-cell Monte Carlo collision (PIC-MCC) simulation. At a fixed pressure of 50 mTorr with the $Ar/O_{2}$ ratio varied from 9:1 to 2:8, the ion energy and angular distributions were collected at the center and edge of the powered electrode. Increasing the oxygen fraction reduced the electron density while enhancing the $O^{-}$ density and electronegativity, driving an electropositive-to-electronegative transition near 8:2, and shifted the dominant positive ion from $Ar^{+}$ to $O_{2}^{+}$, with $O^{+}$ remaining minor owing to charge-exchange loss. The plasma potential and ion-energy peaks generally increased with the oxygen fraction but showed nonmonotonic dependence, while radial edge fields tilted and broadened the angular distributions. To link the ion and oxygen-radical fluxes to $MoS_{2}$ processing without assuming uncertain surface-response coefficients, we interpreted the ion and oxygen-radical fluxes through a phenomenological two-channel surface-reaction scheme, introducing a damaging ion fraction and a radical-to-ion flux ratio. Their opposing trends reveal an intrinsic trade-off, indicating that an intermediate $O_{2}$ fraction offers a more favorable low-damage window than either Ar-rich or strongly oxygen-rich conditions.

## 1. Introduction

As modern semiconductor processing approaches the physical limits of device integration, atomic layer-level precision has become increasingly necessary. Consequently, a substantial portion of key unit processes, including etching, deposition, and surface modification, now relies on plasma-based technologies [1,2,3,4]. In particular, for structures with atomically thin dimensions, such as two-dimensional transition metal dichalcogenides (TMDs) and ultrathin gate dielectrics, the species, energy, flux, and incident angle of ions delivered to the substrate can strongly affect the final process outcomes and device characteristics through defect formation, compositional modification, and interfacial reactions [4,5,6]. Therefore, low-damage plasma processing requires simultaneous control of ion bombardment conditions and reactive neutral fluxes to suppress energetic ion-induced damage while maintaining sufficient chemical reactivity.

$Ar/O_{2}$ mixed plasma has attracted considerable attention as a promising platform for low-damage processing of atomically thin materials [7,8]. This discharge system simultaneously provides physical energy transfer through inert-gas ion bombardment and chemical modification through oxygen radicals and oxygen-containing ions. Experimental studies have demonstrated that plasma patterning or etching of two-dimensional materials such as MoS_2_ can be achieved while maintaining useful material properties [9,10,11]. Despite these successful demonstrations, however, the discharge mechanisms that determine the species-resolved ion flux, oxygen-radical flux, plasma potential, and substrate-incident ion energy and angular distributions remain insufficiently understood. This limitation is particularly important in $Ar/O_{2}$ plasmas because oxygen addition can increase electronegativity through electron attachment, modify the dominant positive ion species, and alter the sheath structure and ion acceleration dynamics [12,13,14,15,16]. Moreover, increasing the oxygen fraction simultaneously modifies the oxygen-radical flux, positive-ion composition, electronegativity, plasma potential, and ion energy distribution. Thus, the low-damage process window cannot be determined from the oxygen fraction alone because enhanced oxygen-assisted chemistry may be accompanied by an increased fraction of energetic ions capable of inducing damage.

Many previous studies have relied on one-dimensional modeling to analyze particle behavior along the axial direction [17,18]. However, in atomically thin TMD processing, low-damage conditions cannot be guaranteed solely by reducing the spatially averaged ion energy. Because the process window between effective material removal or surface modification and plasma-induced damage is narrow, spatial variations in plasma potential, ion flux, ion composition, and ion incident angle can lead to local over-etching, defect generation, or compositional modification [19,20]. Therefore, reliable low-damage processing requires evaluation not only of the averaged plasma behavior near the reactor center but also of the charged-particle dynamics and substrate-incident ion distributions near the electrode edge. Such spatial nonuniformity is important for assessing the robustness of process conditions even in localized test samples and becomes increasingly critical when the process is extended to array-level or large-area TMD patterning [21,22].

In electronegative plasmas, the spatial distribution of negative ions affects electron density, sheath boundary formation, and ion acceleration [12,14,15,18]. Consequently, the density distributions of individual charged species, plasma potential, ion energy distribution functions, and ion angular distribution functions can vary significantly with radial position. These two-dimensional effects cannot be captured by one-dimensional models alone, leaving a critical gap in understanding the relationship between gas mixture-dependent plasma chemistry, ion bombardment, and spatial uniformity in $Ar/O_{2}$-based low-damage plasma processing [19,20,23]. In addition, direct prediction of MoS_2_ surface damage remains challenging because the surface-response coefficients for species-dependent sputtering, oxidation, and defect formation are often uncertain [5,9,10,24,25,26,27]. A flux-based interpretation that distinguishes damaging ion bombardment from oxygen-radical-assisted chemical modification can therefore provide a practical bridge between plasma simulation and process selection.

In this study, we investigate how the $Ar/O_{2}$ mixing ratio modifies the plasma-side conditions governing low-damage ion bombardment and oxygen-assisted surface modification of MoS_2_ in a low-voltage, single-frequency capacitively coupled plasma. The $Ar/O_{2}$ mixing ratio is varied from 9:1 to 2:8 at a fixed pressure of 50 mTorr. Rather than reproducing a specific MoS_2_ etching profile or directly predicting defect densities, the present work is deliberately limited to a plasma-side analysis. The aim is to clarify how oxygen-induced electronegativity affects charged-particle distributions, ion composition, plasma potential, and the energy- and angle-resolved ion flux delivered to the substrate. Direct MoS_2_ plasma–surface reaction coefficients, such as species-dependent sputtering yields and oxidation probabilities, are therefore not specified in this study.

Using GPU-accelerated two-dimensional particle-in-cell Monte Carlo collision simulations, we resolve both the electrode-center and electrode-edge regions and collect substrate-incident ion energy and angular distributions as a function of the gas-mixing ratio [19,20,28]. Furthermore, the simulated ion and oxygen-radical fluxes are interpreted using a phenomenological two-channel surface-reaction picture based on a damaging-ion fraction, $R_{dmg}$, and a radical-to-ion flux ratio, $R_{chem/ion}$. This approach enables assessment of the intrinsic trade-off between radical-assisted chemical modification and energetic-ion-induced damage and provides a physics-based framework for selecting $Ar/O_{2}$ plasma conditions suitable for spatially uniform and low-damage processing of atomically thin TMD materials.

## 2. Simulation Method

**Figure 1:**
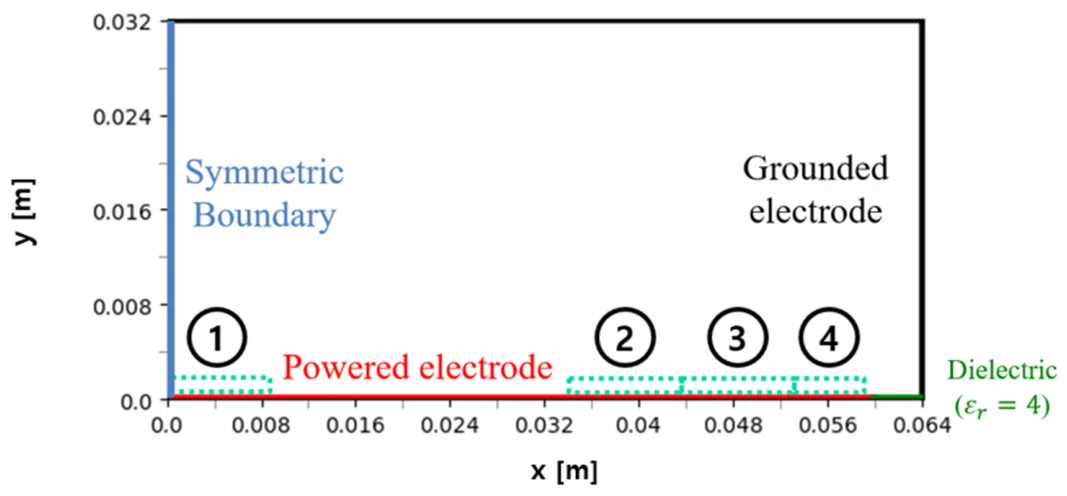
Schematic of the two-dimensional simulation domain. The blue, red, black, and green lines indicate the symmetry boundary, powered electrode, grounded boundaries, and dielectric, respectively. The cyan dashed regions labeled 1–4 denote the surface diagnostic regions used to collect the ion energy distribution functions (IEDFs) and ion angular distribution function (IADFs) at the powered electrode.

The simulation domain and surface diagnostic regions used to evaluate $Ar/O_{2}$ plasma conditions relevant to MoS_2_ patterning are shown in Figure 1. A two-dimensional, three-velocity space electrostatic particle-in-cell/Monte Carlo collision (2D3V PIC-MCC) model was employed to calculate the plasma distribution, species-resolved ion energy distribution functions (IEDFs), and ion angular distribution functions (IADFs) at the powered electrode [19,20]. This section describes the PIC-MCC model, reactor geometry, surface diagnostic method, and the scope of the present simulation.

### 2.1. PIC/MCC Modeling and Simulation Domain

The numerical framework adheres to previously reported PIC-MCC models for capacitively coupled plasmas [28,29].

Figure 1 shows the computational domain and the four surface diagnostic regions used in this study. Because of geometrical symmetry, only half of the reactor was considered; at the symmetry plane, particles are specularly reflected and a Neumann condition ($\frac{\partial \phi }{\partial x}=0\;at\;x\;=0$) was imposed on the potential. The computational domain size was 6.4 cm × 3.2 cm, the grid size was 0.2 mm, and the time step was $1\times 10^{-11}$ s. The bottom electrode, with a length of 6.0 cm, was driven by a sinusoidal RF voltage,

(1) $$V_{\mathrm{rf}}\left(t\right)=V_{0}sin(2\pi f_{0}t),$$

where $V_{0}=100$ V and $f_{0}=50$ MHz. A dielectric with a length of 0.4 cm and a relative permittivity of $\epsilon_{r}$ = 4 was inserted between the powered electrode and the grounded sidewall. The simulation parameters and operating conditions are summarized in Table 1.

### 2.2. Ar/O_2_ Gas Composition and Plasma Chemistry

The total feed-gas pressure was fixed at 50 mTorr for all cases. The $Ar/O_{2}$ mixing ratio was varied by changing the oxygen mole fraction in the feed gas,

(2) $$X_{O_{2}}=\frac{n_{O_{2}}}{n_{Ar}+n_{O_{2}}},$$

from 0.1 to 0.8, corresponding to $Ar/O_{2}$ ratios from 9:1 to 2:8. Here, the pressure refers to the prescribed background feed-gas pressure, $P_{bg}=P_{Ar}+P_{O_{2}}$, rather than the instantaneous sum of all neutral species generated in the plasma.

The background feed gases were ground-state Ar and $O_{2}$. Electrons $Ar^{+}$, $O_{2}^{+}$, $O^{+},$ and $O^{-}$ were treated as kinetic PIC species. Reactive neutral and metastable species, including O(^3^P), O(^1^D), $O_{2}$(a^1^$\Delta_{g})$, $O_{2}(b^{1}\Sigma_{g}$), and $Ar^{*}$, were treated as continuum species. Their densities were updated from production and loss reactions, diffusion transport, and boundary processes such as surface recombination, quenching, and reflection. The $O_{2}$ collisional reactions, together with their cross sections and rate coefficients, were adopted from the work of Gudmundsson et al. [13]. The $Ar/O_{2}$ interaction rate coefficients were taken from research by Lee et al. [17] and Lee and Lieberman [30]. The complete argon reaction set—including the electron-impact, ion-neutral, and metastable reactions, together with their corresponding cross sections and rate coefficients—was adopted from Kim et al. [29].

### 2.3. Surface Diagnostics and Model Scope

Species-resolved ion energy distribution functions (IEDFs) and ion angular distribution functions (IADFs) were collected for $Ar^{+}$, $O_{2}^{+}$, and $O^{+}$ ions incident on the powered electrode. The diagnostic was sampled at four surface regions indicated by the numbered boxes in Figure 1. Region 1 was located near the electrode center, whereas Regions 2–4 were placed progressively closer to the powered-electrode edge. This spatially resolved sampling was used to compare the ion characteristics between the center and edge regions of the powered electrode. For each gas mixture, the IEDFs and IADFs were accumulated over 100 RF cycles after the discharge reached a quasi-steady state.

The MoS_2_ layer itself was not explicitly modeled as a reactive material boundary. Therefore, the simulated data should be interpreted as plasma-side indicators rather than direct predictions of etch rate, profile evolution, oxidation, or defect density. In particular, species-dependent sputtering yields, oxygen-assisted reaction probabilities, and defect-formation cross sections for MoS_2_ are often uncertain or system-specific.

## 3. Results and Discussion

### 3.1. Effects of Gas-Mixture Ratio on Spatial Uniformity and Potential Distribution

#### 3.1.1. Uniformity of Each Species for Various Gas Ratios

**Figure 2:**
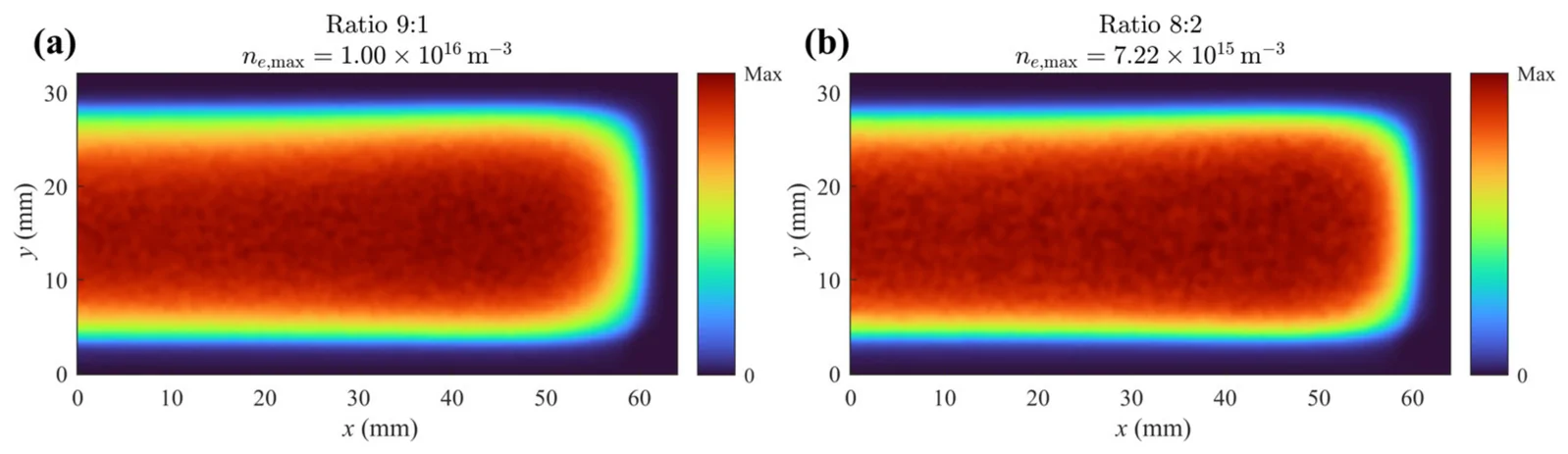

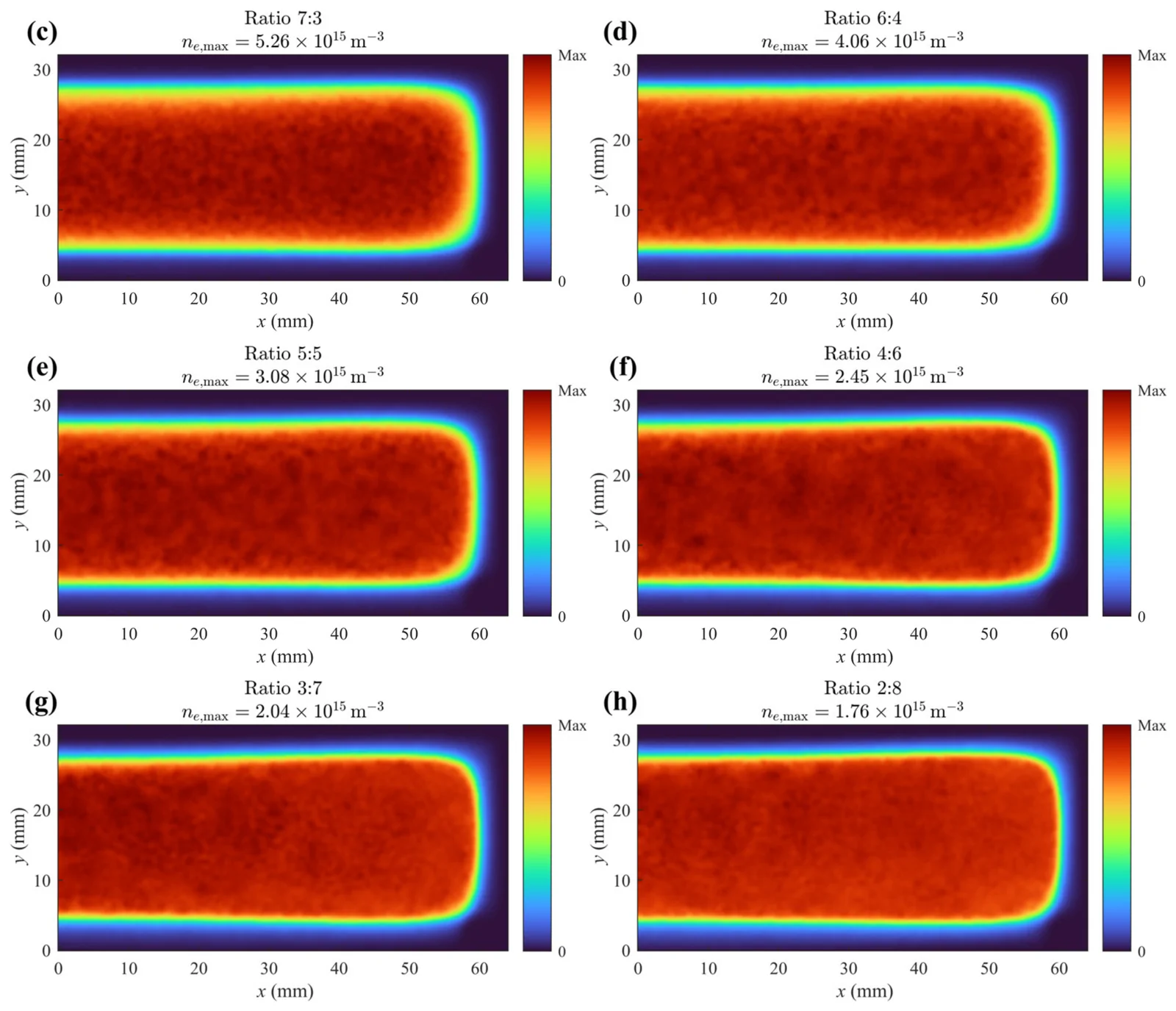
Time-averaged spatial distributions of electron density $n_{e}$ for $Ar/O_{2}$ feed-gas ratios of (**a**) 9:1, (**b**) 8:2, (**c**) 7:3, (**d**) 6:4, (**e**) 5:5, (**f**) 4:6, (**g**) 3:7, and (**h**) 2:8. The maximum electron density, $n_{e,max},$ for each condition is indicated above the corresponding panel.

As shown in Figure 2, the maximum electron density decreases as the oxygen fraction in the $Ar/O_{2}$ feed gas increases. For the $Ar/O_{2}$ = 9:1 condition, corresponding to an oxygen fraction of 10%, the maximum electron density is $1\times 10^{16}\;m^{-3}$ in Figure 2a.

**Figure 3:**
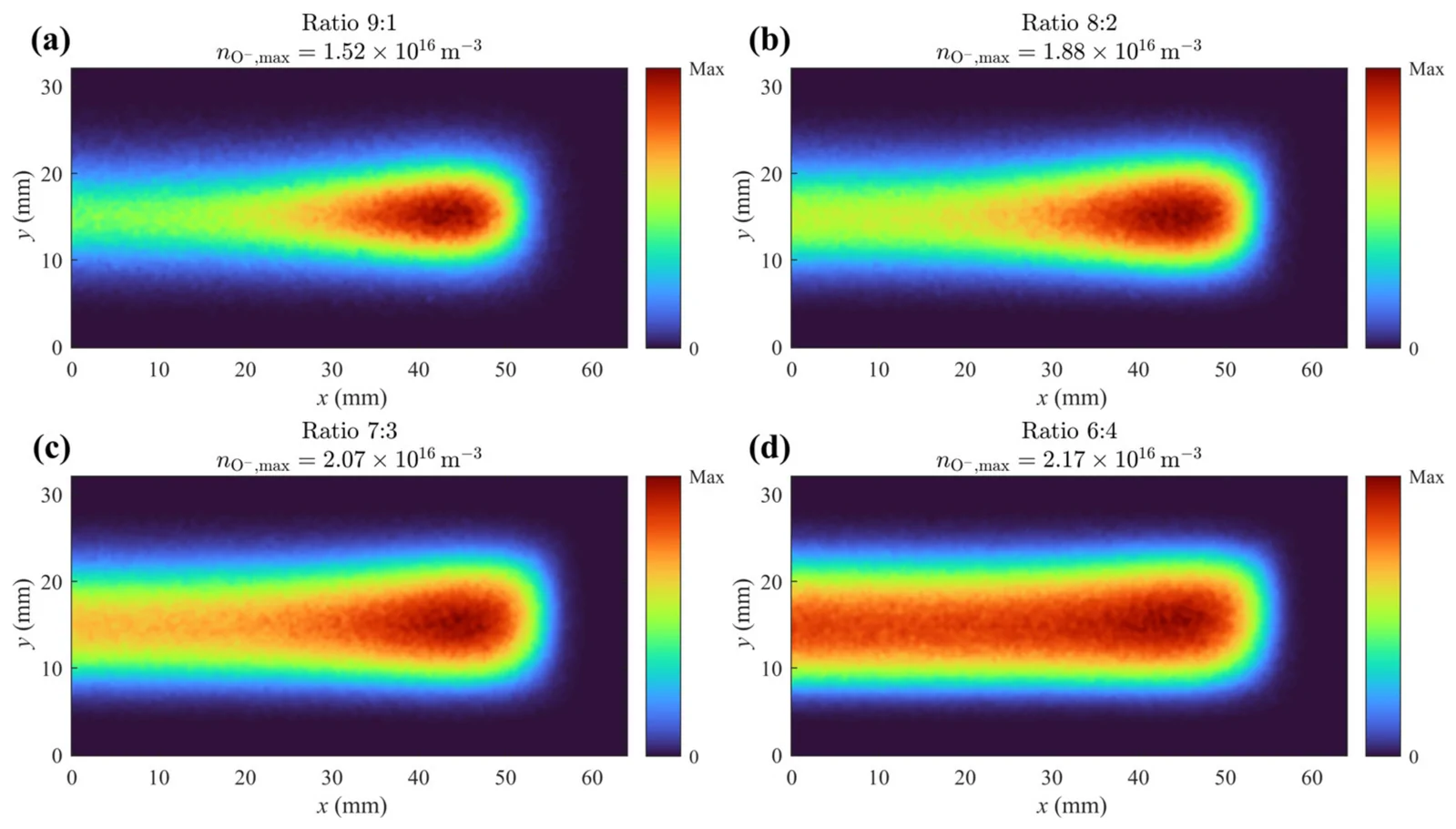

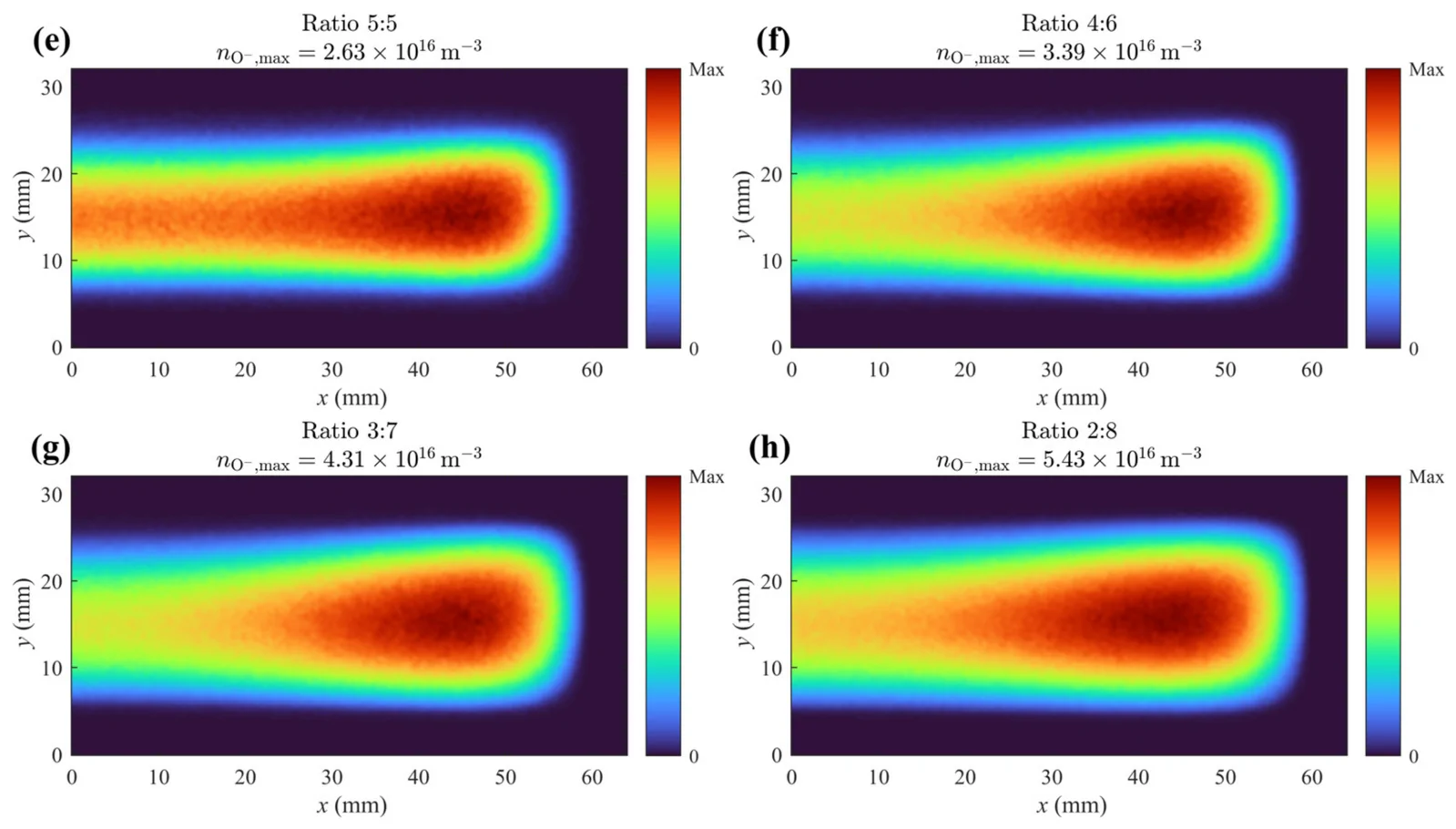
Time-averaged spatial distributions of oxygen negative ion density $n_{O^{-}}$ for $Ar/O_{2}$ feed-gas ratios of (**a**) 9:1, (**b**) 8:2, (**c**) 7:3, (**d**) 6:4, (**e**) 5:5, (**f**) 4:6, (**g**) 3:7, and (**h**) 2:8. The maximum negative ion density, $n_{O^{-},max},$ for each condition is indicated above the corresponding panel.

In contrast, the spatial distributions of oxygen negative ions shown in Figure 3 indicate that the $O^{-}$ density increases with increasing oxygen fraction. The maximum $O^{-}$ density increases from 1.52 $\times \;10^{16}\;m^{-3}$ to 5.43 $\times \;10^{16}\;m^{-3}$, corresponding to an increase by a factor of approximately 3.6. The opposite trends observed in the electron density and oxygen negative-ion density are driven by the same underlying mechanism. As the oxygen fraction in the background gas increases, the frequency of dissociative attachment reactions,

(3) $$e+O_{2}\to O^{-}+O,$$

also increases. Through this reaction, free electrons are consumed while $O^{-}$ ions are simultaneously generated. In addition to this direct loss of electrons through attachment, the increasing $O_{2}$ fraction reduces the electron density through a second mechanism. Unlike argon, $O_{2}$ provides numerous low-threshold inelastic channels—such as vibrational and electronic excitation—that allow electrons to lose energy efficiently in collisions with $O_{2}$ molecules. This enhanced inelastic energy loss cools the high-energy tail of the electron energy distribution, which in turn lowers the electron-impact ionization rate that sustains the discharge. Consequently, the decrease in electron density with increasing $O_{2}$ fraction arises from both an enhanced electron-loss channel (dissociative attachment) and a reduced electron-production channel (ionization), acting together. The interplay between these two trends can be quantified using the electronegativity parameter,

(4) $$\alpha =\frac{n_{O^{-}}}{n_{e}}$$

**Figure 4:**
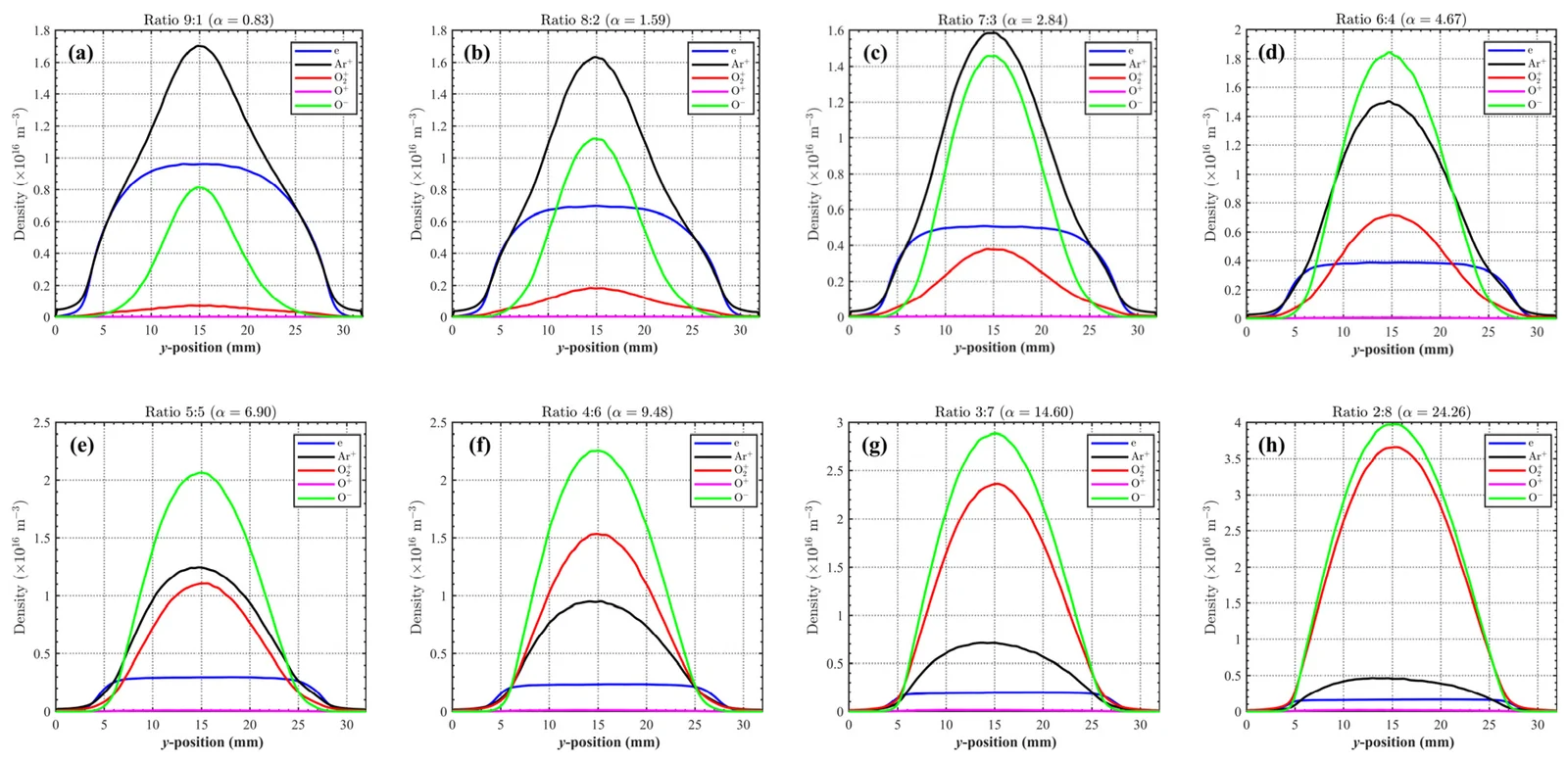
Time-averaged axial density profiles of charged species averaged over the central region (0 ≤x≤16 mm) for $Ar/O_{2}$ feed-gas ratios of (**a**) 9:1, (**b**) 8:2, (**c**) 7:3, (**d**) 6:4, (**e**) 5:5, (**f**) 4:6, (**g**) 3:7, and (**h**) 2:8. The electronegativity parameter, $\alpha =n_{O^{-}}/n_{e}$, evaluated near the plasma bulk center, is indicated above each panel.

Figure 4 shows the time- and space-averaged axial density profiles of charged species along the y-direction for each $Ar/O_{2}$ mixing ratio, averaged over the center region of x = 0–16 mm. The electronegativity parameter evaluated near the plasma bulk center (y = 15 mm) increases monotonically from α=0.83 for the $Ar/O_{2}$ = 9:1 condition to α=24.26 for the $Ar/O_{2}$ = 2:8 condition.

According to the Lichtenberg and Lieberman criterion [15], a plasma is classified as electropositive when α<1 and electronegative when α>1. In the present simulation results, α exceeds unity from the $Ar/O_{2}$ = 8:2 condition (α=1.59), indicating that the transition from electropositive to electronegative plasma begins when the oxygen fraction is 20% or more.

A notable feature of the electronegative plasma is the spatial distribution of $O^{-}$ ions. As shown in Figure 4, for all conditions (a)–(h), $O^{-}$ density reaches its maximum near the plasma bulk center (y ≈ 15 mm), rather than near the electrode surfaces. This behavior occurs because the sheath potential drop barrier accelerates positive ions toward the electrodes while confining negative ions, such as $O^{-}$, within the plasma bulk. Consequently, the accumulation of $O^{-}$ ions modifies the plasma potential structure and can indirectly affect the ion energy distribution functions of ions on the electrode surface, as discussed in detail in Section 3.2.

The dominant positive ion species changes with increasing oxygen fraction. This shift originates from the change in the background neutral composition at fixed total pressure. As the $O_{2}$ fraction increases, the Ar neutral density decreases while the $O_{2}$ neutral density increases. The reduced Ar density, together with the accompanying decrease in electron density, weakens the electron-impact ionization source of $Ar^{+}$, whereas the increased $O_{2}$ density enhances $O_{2}^{+}$ production. As a result, the positive-ion population shifts from an $Ar^{+}$-dominated regime to an $O_{2}^{+}$-dominated one, passing through a mixed regime where the two are comparable. In the $Ar/O_{2}$ = 9:1–7:3 range, $Ar^{+}$ is the dominant ion species, implying that the powered electrode is primarily exposed to $Ar^{+}$-driven physical bombardment. In the $Ar/O_{2}$ = 5:5–4:6 range, $Ar^{+}$ and $O_{2}^{+}$ become comparable, forming a mixed ion-flux regime. At higher oxygen fractions, $O_{2}^{+}$ exceeds $Ar^{+}$, indicating a transition toward an oxygen-rich reactive-ion regime. This species transition changes the relative balance between sputtering-like bombardment and oxygen-assisted surface modification, which is relevant to low-damage MoS_2_ patterning.

**Figure 5:**
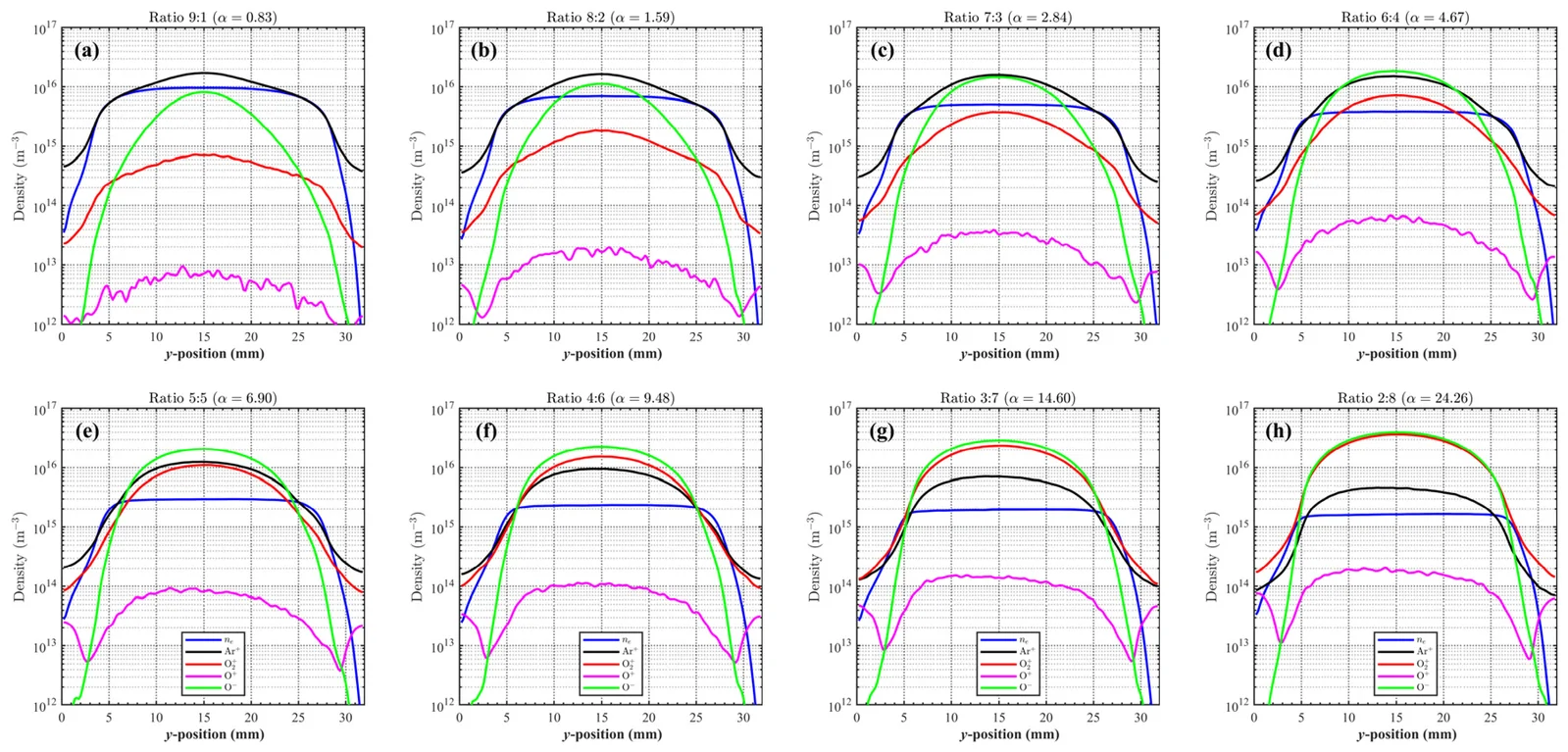
Semi-logarithmic representation of the time-averaged axial density profiles shown in Figure 4 for Ar/O_2_ feed-gas ratios of (**a**) 9:1, (**b**) 8:2, (**c**) 7:3, (**d**) 6:4, (**e**) 5:5, (**f**) 4:6, (**g**) 3:7, and (**h**) 2:8. The logarithmic scale highlights the low-density O^+^ component relative to the dominant charged species.

Figure 5 presents the time-averaged axial density profiles of the charged species in Figure 4 on a semi-logarithmic scale. This representation resolves the $O^{+}$ density, which is almost indistinguishable in the corresponding linear-scale plots. The $O^{+}$ density is on the order of $10^{13}–10^{14}\;m^{-3}$, which is two to three orders of magnitude lower than those of the major ion species. Therefore, $O^{+}$ remains a minor ion component over the investigated range of $Ar/O_{2}$ gas ratios.

**Figure 6:**
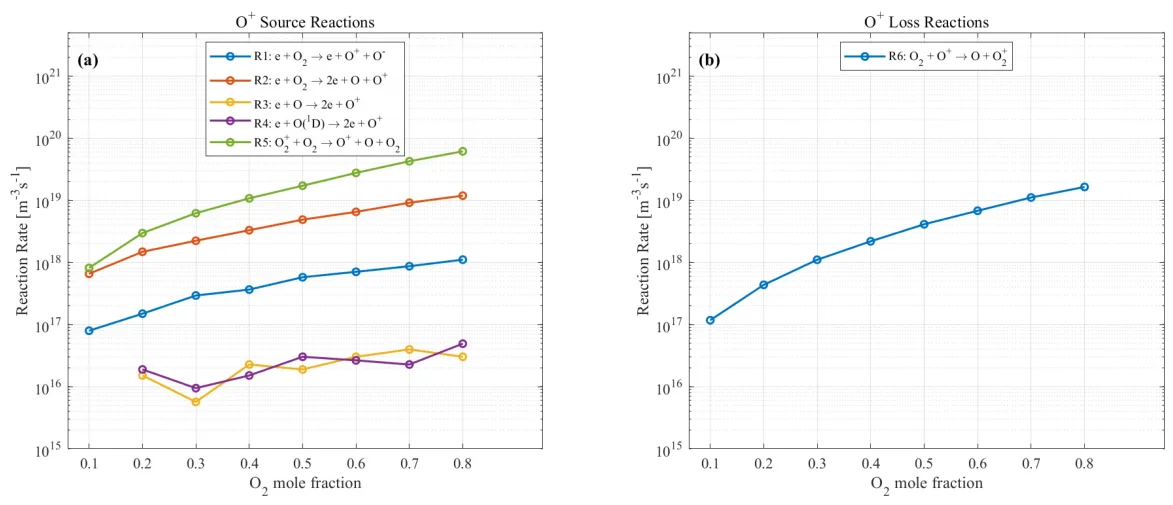
Reaction rates of the major $O^{+}$ reaction pathways as a function of $O_{2}$ mole fraction in the $Ar/O_{2}$ feed gas: (**a**) $O^{+}$ production reactions and (**b**) $O^{+}$ loss reactions.

Figure 6 shows the reaction rate of the major $O^{+}$ production and loss pathways as functions of the $O_{2}$ mole fraction. As shown in Figure 6a, the fragmentation reaction between sheath-accelerated $O_{2}^{+}$ ions and neutral $O_{2}$ molecules,

(5) $$R5:\;O_{2}^{+}+O_{2}\to O^{+}+O+O_{2},$$

exhibits the highest reaction rate among the $O^{+}$ production pathways over the entire $O_{2}$ mole-fraction range. The reaction rate of R5 increases monotonically with the increasing $O_{2}$ fraction, which is consistent with the increase in both the $O_{2}^{+}$ density and the abundance of neutral $O_{2}$ molecules. However, electron-impact dissociative ionization of $O_{2}$,

(6) $$R2:\;e+O_{2}\to 2e+O+O^{+},$$

also provides a substantial contribution to $O^{+}$ production. Its reaction rate remains within the same order of magnitude as that of R5 over most of the investigated $O_{2}$ mole-fraction range. Therefore, R5 and R2 can be regarded as the two major $O^{+}$ production channels. In contrast, the other production pathways, such as R1, R3, and R4, exhibit much lower reaction rates and make only minor contributions to $O^{+}$ generation.

As shown in Figure 6b, the dominant $O^{+}$ loss pathway is charge exchange with $O_{2}$:

(7) $$R6:\;O_{2}+O^{+}\to O+O_{2}^{+}$$

The reaction rate of R6 increases rapidly with increasing $O_{2}$ fraction, indicating that $O^{+}$ ions are efficiently lost through charge exchange with $O_{2}$ molecules. This efficient charge-exchange loss converts $O^{+}$ into $O_{2}^{+}$ and accounts for the relatively low $O^{+}$ density maintained in the bulk plasma.

Overall, these results indicate that $O^{+}$ ions are produced mainly through two pathways: fragmentation of $O_{2}^{+}$ by collision with $O_{2}$ and electron-impact dissociative ionization of $O_{2}$. Although the fragmentation reaction associated with sheath-accelerated $O_{2}^{+}$ ions offers the largest production rate, the electron-impact dissociative ionization pathway also remains significant. Nevertheless, because the $O^{+}$ density is substantially lower than those of $Ar^{+}$ and $O_{2}^{+}$ and because $O^{+}$ is efficiently consumed by charge exchange with $O_{2}$, $O^{+}$ is expected to make only a minor contribution to the total ion flux incident on the electrode. Accordingly, its direct contribution to ion-induced surface modification is likely limited.

#### 3.1.2. Plasma Potential Distribution as a Function of the Gas-Mixing Ratio

**Figure 7:**
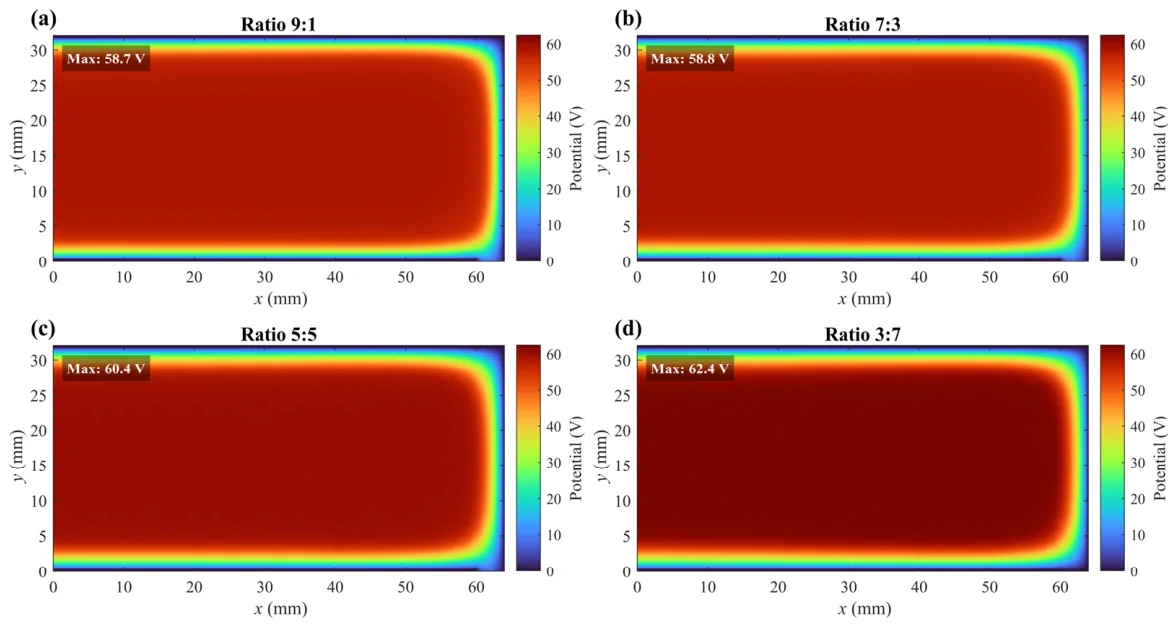
Time-averaged spatial distribution of plasma potential for representative $Ar/O_{2}$ feed gas ratios of (**a**) 9:1, (**b**) 7:3, (**c**) 5:5, (**d**) 3:7. The maximum plasma potential for each condition is indicated in the corresponding panel.

Figure 7a–d show the time-averaged 2D plasma potential distributions for four representative $Ar/O_{2}$ feed-gas ratios. In all cases, the plasma bulk maintains a relatively uniform high potential, while a pronounced sheath potential drop is formed near the electrode surfaces. This indicates that the overall potential structure remains similar across the gas-mixing conditions, although the potential magnitude and sheath characteristics vary with the oxygen fraction.

**Figure 8:**
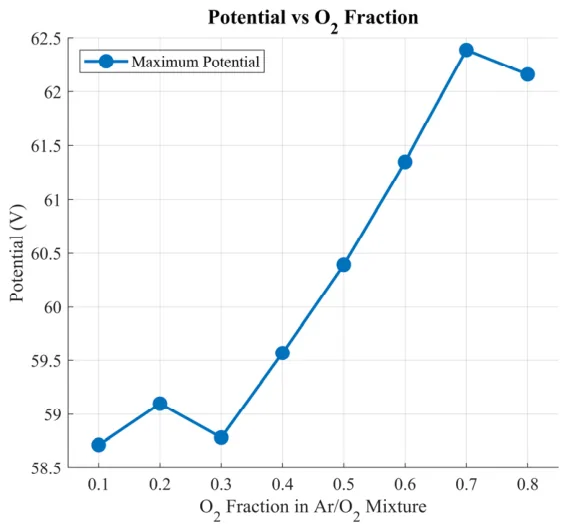
Maximum time-averaged plasma potential as a function of $O_{2}$ mole fraction in the $Ar/O_{2}$ feed gas.

Figure 8 shows the variation in the maximum time-averaged plasma potential as a function of the $O_{2}$ mole fraction in the $Ar/O_{2}$ mixture. The maximum plasma potential generally increases with the oxygen fraction. This trend can be attributed to the enhanced electronegativity of the discharge. As the $O_{2}$ fraction increases, electron attachment and dissociative attachment reactions consume free electrons and generate negative oxygen ions, particularly $O^{-}\;$. The resulting reduction in electron density and modification of the charged-particle balance lead to an increase in the plasma potential required to sustain the discharge and balance charged-particle losses to the boundaries [16]. More specifically, the plasma potential is established to confine the highly mobile electrons and thereby equalize the electron and positive-ion losses to the boundaries. As the electron density decreases and an increasing share of the negative charge is carried by the slow, well-confined $O^{-}$ ions, maintaining ambipolar balance requires a larger bulk-to-wall potential drop, so the bulk plasma potential rises as the discharge becomes more electronegative.

However, the potential does not increase strictly monotonically: slight decreases appear when $X_{O_{2}}$ increases from 0.2 to 0.3 and from 0.7 to 0.8. Since the maximum plasma potential is governed not by the electronegativity itself but by the underlying electron energy distribution and the charged-particle balance that sustain the discharge, a strictly monotonic dependence on gas composition is not expected. Notably, these local decreases coincide with the composition ranges where the discharge structure is reorganized: the decrease near $X_{O_{2}}$ = 0.2–0.3 occurs just after the onset of the electropositive-to-electronegative transition (α exceeding unity at $X_{O_{2}}$ = 0.2), and the decrease near $X_{O_{2}}$ = 0.8 occurs in the strongly electronegative regime (α ≈ 24), where the bulk approaches an ion–ion plasma. Consistently, the electron-impact ionization rates of atomic oxygen (R3 and R4 in Figure 6a) also exhibit a local minimum near $X_{O_{2}}$ = 0.3, indicating that the electron energy distribution is locally modified in this composition range. These observations suggest that the nonmonotonic plasma potential reflects the restructuring of the discharge across the electronegative transition rather than a simple monotonic dependence on the $O_{2}$ fraction. Therefore, the plasma potential in $Ar/O_{2}$ discharges should not be estimated by simple linear interpolation with gas composition; instead, it should be evaluated for each mixing condition when designing low-damage plasma processes for MoS_2_ patterning.

**Figure 9:**
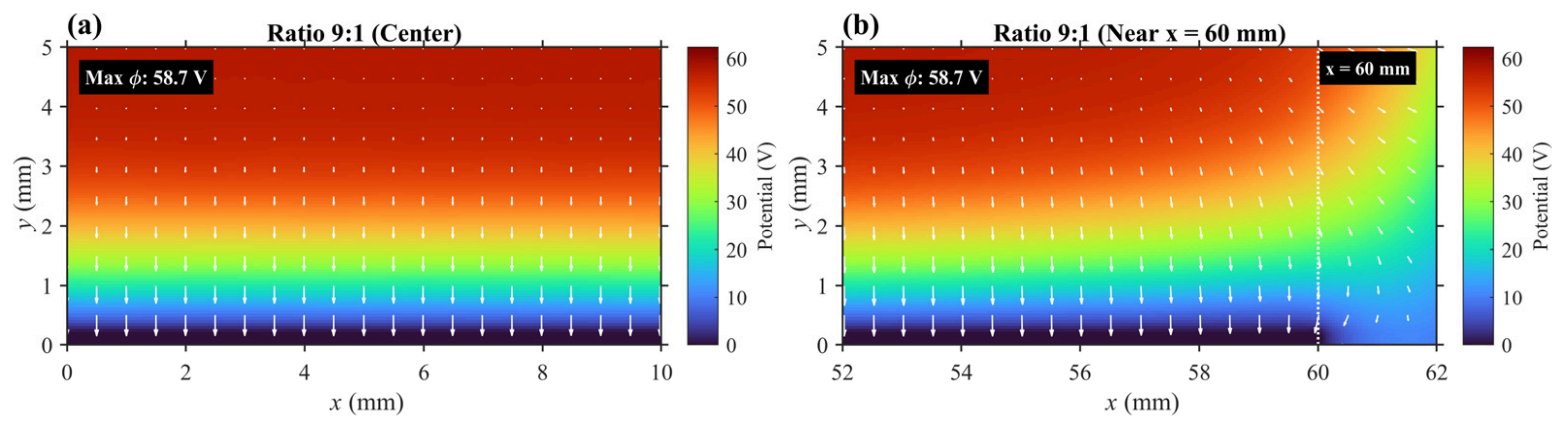

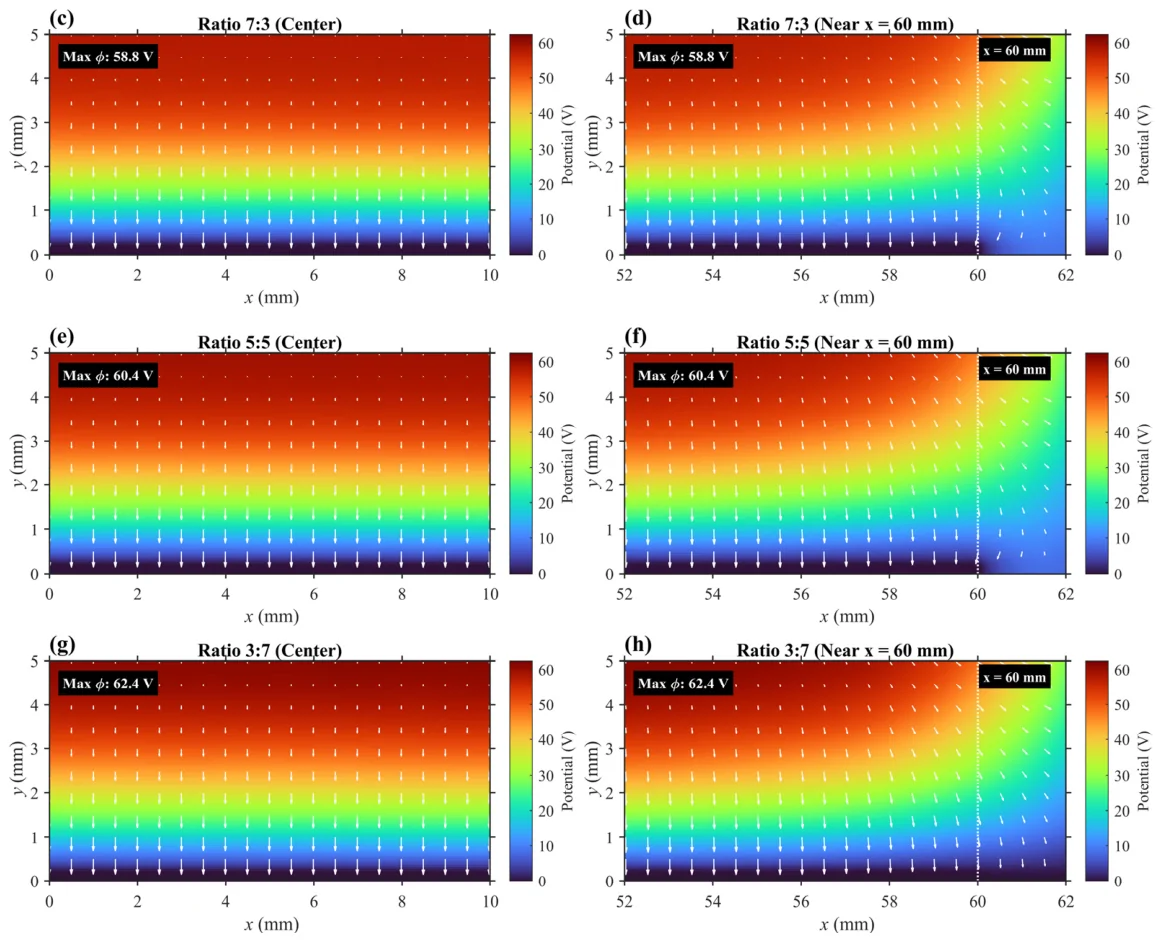
Time-averaged plasma potential distribution and electric-field vectors near the powered-electrode center and edge for representative $Ar/O_{2}$ feed-gas ratios: (**a**,**b**) 9:1, (**c**,**d**) 7:3, (**e**,**f**) 5:5, and (**g**,**h**) 3:7. The left column shows the electrode-center region (0 ≤x≤10 mm), and the right column shows the electrode-edge region (50≤x≤60 mm).

Figure 9a–h compare the time-averaged two-dimensional plasma potential distribution and electric-field vectors near the powered-electrode center (0≤x≤10 mm) and edge $\left(50\leq x\leq 60\;\mathrm{mm}\right)$ for representative $Ar/O_{2}$ feed-gas ratios. In the electrode-center region, although the potential level and sheath potential drop vary with the gas-mixing ratio, the electric-field vectors remain mostly aligned with the surface-normal direction. This indicates that in the center region, changes in gas composition mainly affect the incident ion energy rather than the incident ion angle.

In contrast, near the powered-electrode edge, the lateral potential gradient becomes significant because of the combined influence of the sidewall and electrode-edge boundary conditions. As a result, a radial component is generated in the electric field, and the electric-field vectors deviate from the surface-normal direction. The radial component of the electric field generated near the electrode edge can deflect ion trajectories away from the surface-normal direction.

This edge-induced ion tilting is directly related to process uniformity because it changes the local ion energy and angular distributions at the powered electrode surface. While ions in the center region are accelerated mainly along the surface-normal direction, the radial electric-field component near the electrode edge can produce non-normal ion incidence. As a result, the edge region may experience ion exposure conditions that differ from those at the electrode center, leading to spatial nonuniformity during MoS_2_ patterning. Therefore, reducing the radial electric-field component near the electrode edge is important for improving the uniformity of low-damage plasma processing.

### 3.2. Surface Ion Energy and Angular Distribution as a Function of the Gas-Mixing Ratio

#### 3.2.1. Ion Energy and Angular Distribution at the Powered Electrode

**Figure 10:**
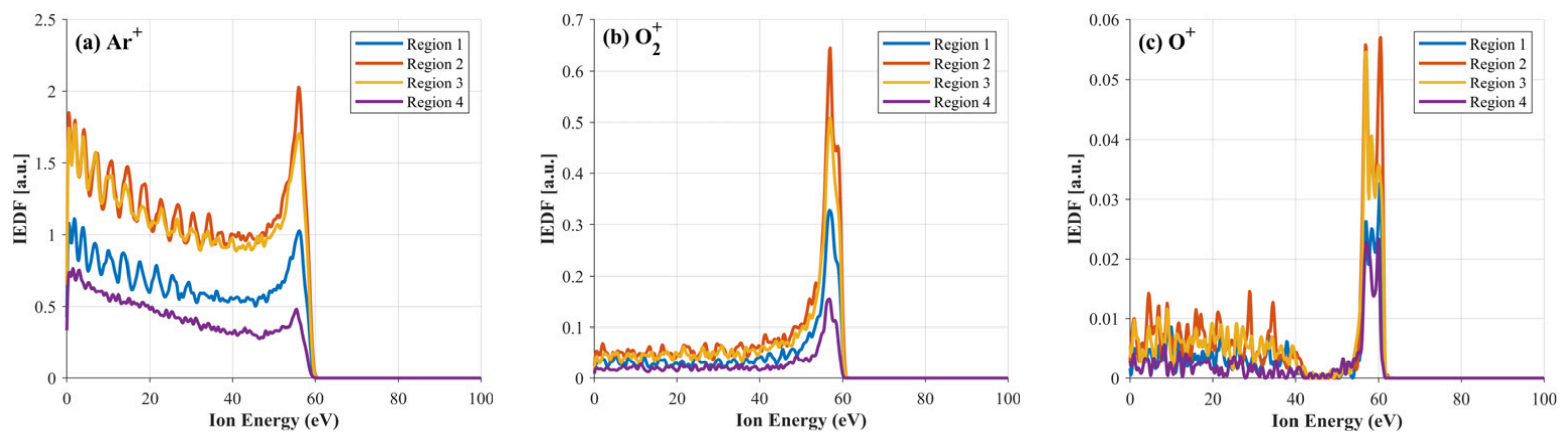
Time-averaged ion energy distributions functions (IEDFs) of (**a**) $Ar^{+}$, (**b**) $O_{2}^{+}$, and (**c**) $O^{+}$ ions incident on the powered electrode for the $Ar/O_{2}$ = 9:1 condition. The distributions were collected at surface diagnostic regions 1–4 indicated in Figure 1 and accumulated over 100 RF cycles.

Figure 10a–c show the time-averaged ion energy distribution functions (IEDFs) of ions incident on the powered electrode for $Ar/O_{2}$ = 9:1 condition. The distributions were accumulated over 100 RF cycles and compared for $Ar^{+},O_{2}^{+},$ and $O^{+}$ ions collected at four surface diagnostic regions defined in Figure 1. Regions 1 and 2 are located away from the electrode edge; therefore, their IEDF shapes are generally similar. The difference in the absolute magnitude between these two regions is mainly attributed to the difference in the ion collection area. For $Ar^{+}$ ions, a dominant high-energy peak appears at approximately 56 eV. This peak is attributed to ion acceleration across the sheath potential drop, which gives the ions an energy close to q$\Delta V_{sheath}$. In addition to this high-energy peak, several small peaks and tail structures are observed in the low-energy range. These features indicate that some ions undergo ion-neutral collisions inside the sheath at 50 mTorr, particularly charge-exchange collisions or elastic scattering, and reach the electrode surface without gaining the sheath potential drop energy. Therefore, unlike the narrow single-peak IEDF expected in a collisionless sheath, the present result reflects the characteristics of a collisional sheath.

For $O_{2}^{+}$ and $O^{+}$ ions, the main high-energy peaks are also located near 56 eV, similar to $Ar^{+}$. However, the overall magnitude of the $O^{+}$ distribution is much smaller than that of the other ion species, indicating that the $O^{+}$ population incident on the electrode is relatively low under the $Ar/O_{2}$ = 9:1 condition. The high-energy peak position does not change significantly between the inner and edge regions. Nevertheless, Region 4 exhibits a lower-incident ion population than the other regions with comparable collection areas. In addition, whereas the high-energy component is dominant in the inner regions, the low-energy component becomes more pronounced near the electrode edge. It suggests that the edge sheath structure and collisional ion transport reduce the effective ion acceleration in the edge region.

It should be noted that the bimodal or multimodal structure of an IEDF does not arise solely from ion-neutral collisions within the sheath. Even in a collisionless sheath, the shape of the IEDF is governed by the ratio of the ion transit time across the sheath, $\tau_{ion}$, to the RF period, $\tau_{RF}$ [31,32]. When $\tau_{ion}/\tau_{RF}\;\ll 1$, an ion traverses the sheath within a small fraction of the RF cycle and therefore experiences an essentially frozen, instantaneous sheath field; ions entering at different RF phases are accelerated by different instantaneous voltages, which produces two peaks near the high-energy edge separated by an energy splitting that scales with the amplitude of the RF sheath-voltage modulation. In the opposite limit, $\tau_{ion}/\tau_{RF}\gg 1$, an ion crosses the sheath over many RF cycles and responds only to the time-averaged field, yielding a single narrow peak. Because $\tau_{Ion}$ scales with the square root of the ion mass, this transit-time effect also contributes to the mass dependence of the distributions, with lighter ions exhibiting a more pronounced high-energy modulation than heavier ones.

In the present simulations, the two mechanisms dominate different regions of the energy spectrum. The low-energy component, which forms a continuum extending from zero energy up to the main peak, is attributed to charge-exchange and scattering collisions within the sheath. In contrast, the structure near the high-energy main peak reflects the finite $\tau_{ion}/\tau_{RF}$ ratio, through which collisionless RF modulation broadens and splits the high-energy edge of the distribution. The observed IEDFs therefore result from the combined action of collisional low-energy transport and transit-time-governed high-energy modulation.

**Figure 11:**
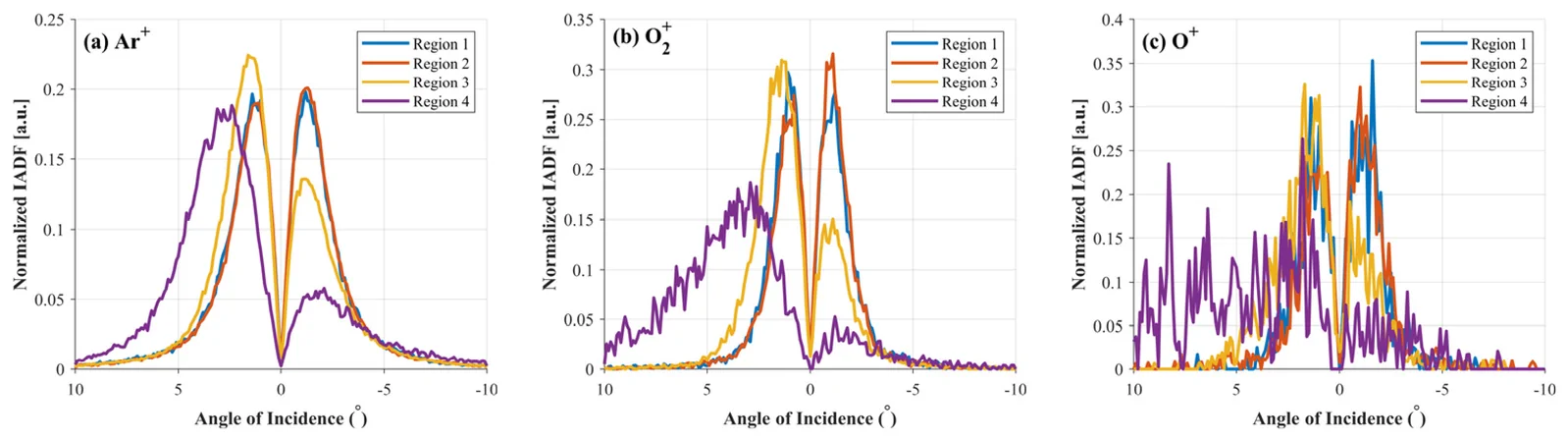
Normalized ion angular distribution functions (IADFs) of (**a**) $Ar^{+}$, (**b**) $O_{2}^{+}$, and (**c**) $O^{+}$ ions incident on the powered electrode for the $Ar/O_{2}$ = 9:1 condition. The incident angle is defined with respect to the surface normal, and the distributions were sampled at the surface diagnostic regions 1–4 shown in Figure 1.

Figure 11a–c show the normalized ion angular distribution functions (IADFs) obtained for the same $Ar/O_{2}$ = 9:1 condition. In Regions 1 and 2, all three ion species exhibit relatively narrow peaks near the surface-normal direction, indicating that ions accelerated in the sheath are incident almost vertically onto the electrode surface. In Region 3, near-normal incidence remains dominant, but the angular distribution becomes slightly asymmetric, suggesting an increased contribution of ions tilted toward the electrode edge. In contrast, in region 4, which is located closest to the electrode edge, the peak position shifts noticeably and the angular spread increases. This indicates that non-normal ion incidence becomes more significant near the edge. This trend is consistent with the two-dimensional potential distribution discussed above: the lateral potential gradient near the edge generates a radial electric-field component, which deflects ion trajectories away from the surface-normal direction. Because this behavior is commonly observed for the positive ion species, the tilted incidence near the edge can be interpreted as a general sheath ion-dynamics effect induced by the edge electric field. For $O^{+}$ ions, the distribution contains larger noise because the number of ions incident on the surface is relatively small under the 9:1 condition.

The incident angle φ is defined with respect to the surface normal of the powered electrode. A positive φ corresponds to ions whose lateral velocity component points from the center toward the electrode edge (sidewall), while a negative φ corresponds to ions directed from the edge toward the symmetry plane at the center.

**Figure 12:**
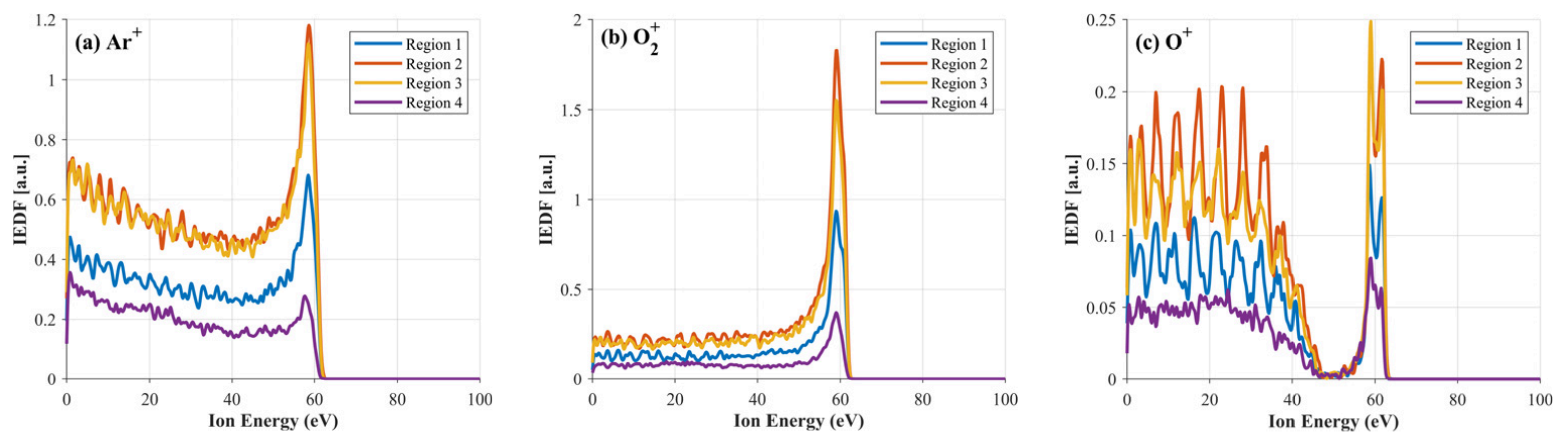
Time-averaged ion energy distribution functions (IEDFs) of (**a**) $Ar^{+}$, (**b**) $O_{2}^{+}$, and (**c**) $O^{+}$ ions incident on powered electrode for the $Ar/O_{2}$ = 5:5 condition. The distributions were collected at surface diagnostic regions 1–4 indicated in Figure 1 and accumulated over 100 RF cycles.

Figure 12a–c show the time-averaged IEDFs for the $Ar/O_{2}$ = 5:5 condition. As discussed in the plasma potential analysis, increasing the oxygen fraction generally increases the maximum plasma potential. This increase leads to a shift in the main IEDF peaks toward higher energies compared with the $Ar/O_{2}$ = 9:1 condition. For $Ar^{+}$ ions, the low-energy component becomes less dominant relative to the high-energy peak, suggesting that the collisional formation of slow $Ar^{+}$ ions within the sheath becomes less pronounced as the Ar fraction decreases. For $O_{2}^{+}$ ions, a strong high-energy peak is observed at a similar energy position to that of $Ar^{+}$. In the case of $O^{+}$ ions, the absolute incident population remains the smallest among the three ion species, but the peak position is comparable to those of $Ar^{+}$ and $O_{2}^{+}$. In the low-energy region, the $O^{+}$ distribution shows a structure similar to that of $Ar^{+}$. This behavior can be understood by considering that $O^{+}$ ions are partly generated through fragmentation reactions inside the sheath and are subsequently accelerated from their formation positions toward the electrode surface.

**Figure 13:**
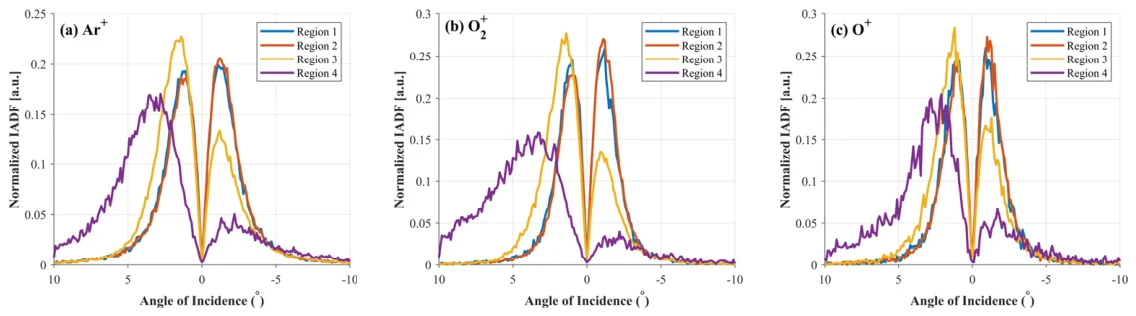
Normalized ion angular distribution functions (IADFs) of (**a**) Ar^+^, (**b**) O^2+^, and (**c**) O^+^ ions incident on the powered electrode for the Ar/O_2_ = 5:5 condition. The incident angle is defined with respect to the surface normal, and the distributions were sampled at the surface diagnostic regions 1–4 shown in Figure 1.

Figure 13a–c show the normalized IADFs for the $Ar/O_{2}$ = 5:5 condition. Compared with the $Ar/O_{2}$ = 9:1 case, the region-dependent angular behavior remains nearly unchanged. It indicates that changes in gas composition mainly modify the ion energy distribution via the plasma potential and the sheath potential drop. In contrast, the two-dimensional sheath geometry near the electrode edge more strongly governs the angular distribution. In the case of $O^{+}$ ions, the incident population is higher than that in 9:1 condition because of the increased oxygen fraction. As a result, the statistical noise in the $O^{+}$ IADF is reduced, and the same region-dependent trend observed for $Ar^{+}$, and $O_{2}^{+}$ becomes clearer. Specifically, narrow near-normal peaks form in the inner regions, while tilted, broadened angular distributions appear near the edge. This result supports the idea that the edge-induced angular broadening and tilting are not specific to a single ion species but are a common consequence of the radial electric-field component near the powered-electrode edge.

**Figure 14:**
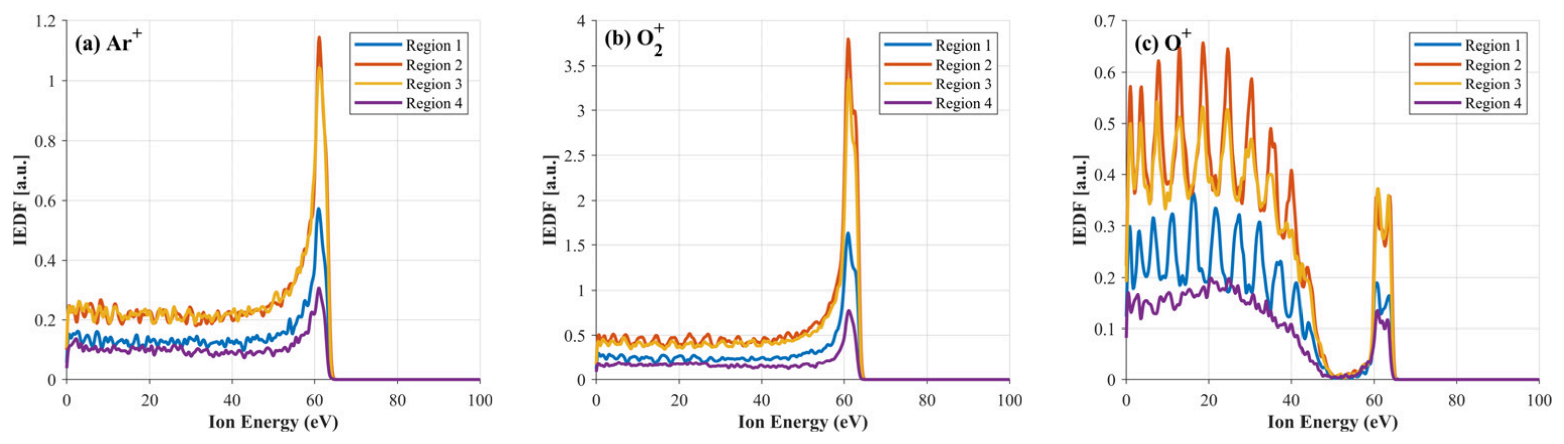
Time-averaged ion energy distribution functions (IEDFs) of (**a**) $Ar^{+}$, (**b**)$\;O_{2}^{+}$, and (**c**) $O^{+}$ ions incident on the powered electrode for the $Ar/O_{2}$ = 2:8 condition. The distributions were collected at surface diagnostic regions 1–4 indicated in Figure 1 and accumulated over 100 RF cycles.

Figure 14a–c show the time-averaged IEDFs for the oxygen-rich $Ar/O_{2}$ = 2:8 condition. The shift in the IEDF peak energy associated with the increased $O_{2}$ mole fraction is also observed in this case. As the Ar fraction decreases, the IEDF shapes of $Ar^{+}$ and $O_{2}^{+}$ become more similar, indicating that both ion species experience comparable sheath acceleration under the oxygen-rich plasma condition. In contrast, $O^{+}$ ions show markedly different low-energy behavior. Because the $O_{2}$ density is high in the 2:8 mixture, $O^{+}$ ions have an increased probability of undergoing scattering or charge transfer-related collisions with $O_{2}$ molecules inside the sheath. Consequently, the low-energy incident population becomes larger than the high-energy component for $O^{+}$ ions. This result indicates that $O^{+}$ bombardment under oxygen-rich conditions is more strongly affected by collisional sheath transport than the bombardment of $Ar^{+}$ or $O_{2}^{+}$ ions. Overall, these results show that the $Ar/O_{2}$ mixing ratio changes not only the dominant positive ion species but also the energy-resolved and angle-resolved ion bombardment at the powered electrode. The increase in oxygen fraction enhances oxygen ion-related bombardment, while the electrode-edge field introduces angular nonuniformity that is largely independent of ion species. These trends are important for designing low-damage MoS_2_ patterning conditions because the balance between directional ion bombardment, low-energy collisional ions, and oxygen-assisted surface modification can influence the spatial uniformity of plasma processing.

**Figure 15:**
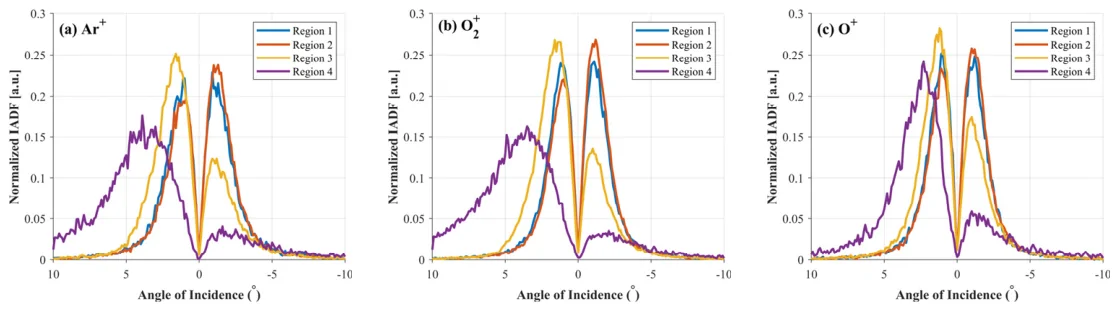
Normalized ion angular distribution functions (IADFs) of (**a**) $Ar^{+}$, (**b**)$\;O_{2}^{+}$, and (**c**) $O^{+}$ ions incident on the powered electrode for the $Ar/O_{2}$ = 2:8 condition. The incident angle is defined with respect to the surface normal, and the distributions were sampled at the surface diagnostic regions 1–4 shown in Figure 1.

Figure 15a–c show the normalized IADFs for the Ar: $O_{2}$ = 2:8 condition. Compared with the previous cases, the $O_{2}^{+}$, distribution shows no significant change, whereas the $O^{+}$ distribution exhibits an increased population in the near-normal range (approximately 0–5°), indicating that a larger fraction of $O^{+}$ ions is incident nearly perpendicular to the electrode surface. In contrast, the $Ar^{+}$ distribution becomes broader, with its near-normal peak reduced and the angular spread increased. Since the incident $Ar^{+}$ population remains sufficiently large, this broadening represents a genuine physical effect rather than a statistical artifact. It is consistent with the enhanced collisional transport of $Ar^{+}$ under the oxygen-rich condition: as discussed for the IEDFs, the high $O_{2}$ density increases the probability of scattering and charge-transfer collisions experienced by $Ar^{+}$ within the sheath, which not only enlarges the low-energy component of its IEDF but also broadens its angular distribution by randomizing the transverse ion velocity.

#### 3.2.2. Flux-Based Assessment of the Damage–Chemistry Trade-Off in MoS_2_ Patterning

To relate the simulated plasma fluxes to $MoS_{2}$ processing without fitting unknown surface-response coefficients such as sputtering yields or reaction probabilities, we note that the present simulation resolves the plasma environment rather than the etching process itself; to connect the two, we interpret the simulated ion and radical fluxes in terms of the phenomenological multistep surface reaction scheme proposed by Lee et al. for $O_{2}/Ar$ plasma etching of $MoS_{2}\to MoS\to Mo,$ with two parallel channels acting at each sulfur-removal step. Energetic positive ions $\left(O_{2}^{+},O^{+},Ar^{+}\right)$ remove sulfur by momentum transfer, whereas reactive oxygen radicals convert surface sulfur into volatile $SO_{2}:$

(8) $$MoS_{2}\left(s\right)\overset{O_{2}^{+},O^{+},Ar^{+}}{\to }\begin{array}{c} \mathrm{MoS}(s)+S \end{array}$$

(9) $$MoS_{2}\left(s\right)+2O\to MoS\left(s\right)+SO_{2}$$

(10) $$MoS\left(s\right)\overset{O_{2}^{+},O^{+},Ar^{+}}{\to }Mo\left(s\right)+S$$

(11) $$MoS\left(s\right)+2O\to Mo\left(s\right)+SO_{2}$$

(12) $$Mo\left(s\right)\overset{O_{2}^{+},O^{+},Ar^{+}}{\to }Mo(g)$$

The species above the arrows denote ion-induced physical removal, and the +2O reactions denote oxygen-assisted chemical removal. Reactions R8, R10, and R12 constitute the ion-driven channel, which depends on the incident-ion energy and is associated with sputtering, sulfur-vacancy formation, and, at sufficiently high energy, lattice damage; consistent with this, an increase in ion-bombardment energy is known to drive desulfurization of the $MoS_{2}$ surface [9]. Reactions R9 and R11 constitute the oxygen-assisted chemical channel, which removes sulfur with comparatively little energetic bombardment; we treat this channel in a simplified form, leaving the detailed surface energetics outside the present scope.

Within this scheme, the $MoS_{2}$ removal rate separates into an ion bombardment term and a chemical term,

(13) $$R_{etch}\approx Y_{s}\Gamma_{+}+\gamma_{0}\Gamma_{O}$$

where $Y_{s}$ is an effective sputtering yield and $\gamma_{0}$ an effective reaction probability. These coefficients are not known a priori, because they depend on the $MoS_{2}$ surface state, ion energy, incidence angle, oxygen coverage, and defect configuration; indeed, even in the original formulation they were not fixed to absolute values ($Y_{s}$ normalized to unity and $\gamma_{0}$ treated as a free parameter over $10^{\text{–}3}-10^{-2}$), yielding only a relative etch rate [9]. The ion and radical fluxes, however, are obtained directly from our simulation and are inaccessible to the global model. We quantify the ion-induced damage propensity through the energy-resolved, threshold-exceeding ion fraction $R_{dmg}$, and the relative chemical supply through the radical-to-ion flux ratio $R_{chem/ion}$, without assuming specific values of $Y_{s}$ or $\gamma_{0}$.

Both fluxes are evaluated at the electrode. For each ion species s∈ $\left\{Ar^{+},O^{+},O_{2}^{+}\right\}$, the total flux is obtained from $\Gamma_{\mathrm{ion},s}=<n_{s}u_{s,y}{>}_{electrode},$ and its energy dependence from the corresponding normalized distribution $f_{\mathrm{IEDF},s}(E)$, with $\int_{0}^{\infty }f_{\mathrm{IEDF},s}\left(E\right)dE=1$. The oxygen-radical flux is estimated as the thermal effusion flux of each atomic-oxygen state $j\in \left\{O\left(3P\right),O\left(1D\right)\right\}$,

(14) $$\Gamma_{O,j}=\frac{1}{4}n_{O,j}{\bar{v}}_{O,j},\;{\bar{v}}_{O,j}=\sqrt{\frac{8k_{B}T_{g}}{\pi m_{O}}}$$

where $n_{O,j}$ is the near-electrode radical density and $T_{g}$ is the neutral temperature.

The damaging-ion fraction is defined as the fraction of incident ions whose kinetic energy exceeds a selected damage threshold, $E_{\mathrm{th}}:$

(15) $$R_{\mathrm{dmg}}\left(E_{\mathrm{th}}\right)=\frac{\Gamma_{\mathrm{ion}}\left(E>E_{\mathrm{th}}\right)}{\Gamma_{\mathrm{ion},\mathrm{total}}}=\frac{\sum_{s}\Gamma_{\mathrm{ion},s}\int_{E_{\mathrm{th}}}^{\infty }f_{\mathrm{IEDF},s}\left(E\right)\;dE}{\sum_{s}\Gamma_{\mathrm{ion},s}}$$

We adopt $E_{\mathrm{th}}$ = 50 eV, the energy near which $Ar^{+}$ bombardment has been experimentally shown to begin generating sulfur vacancies in $MoS_{2}$, whereas removal of the underlying Mo and destruction of the layer require energies near 100 eV [33]. $R_{dmg}$ should therefore be read as a threshold-exceeding ion fraction associated with the onset of sulfur-vacancy generation, rather than as an absolute defect density. This threshold is conservative: for the first sulfur-removal step, ab initio calculations place the intrinsic sputtering threshold of pristine $MoS_{2}$ near 31 eV, dropping to 14 eV under oxygen functionalization [33].

**Figure 16:**
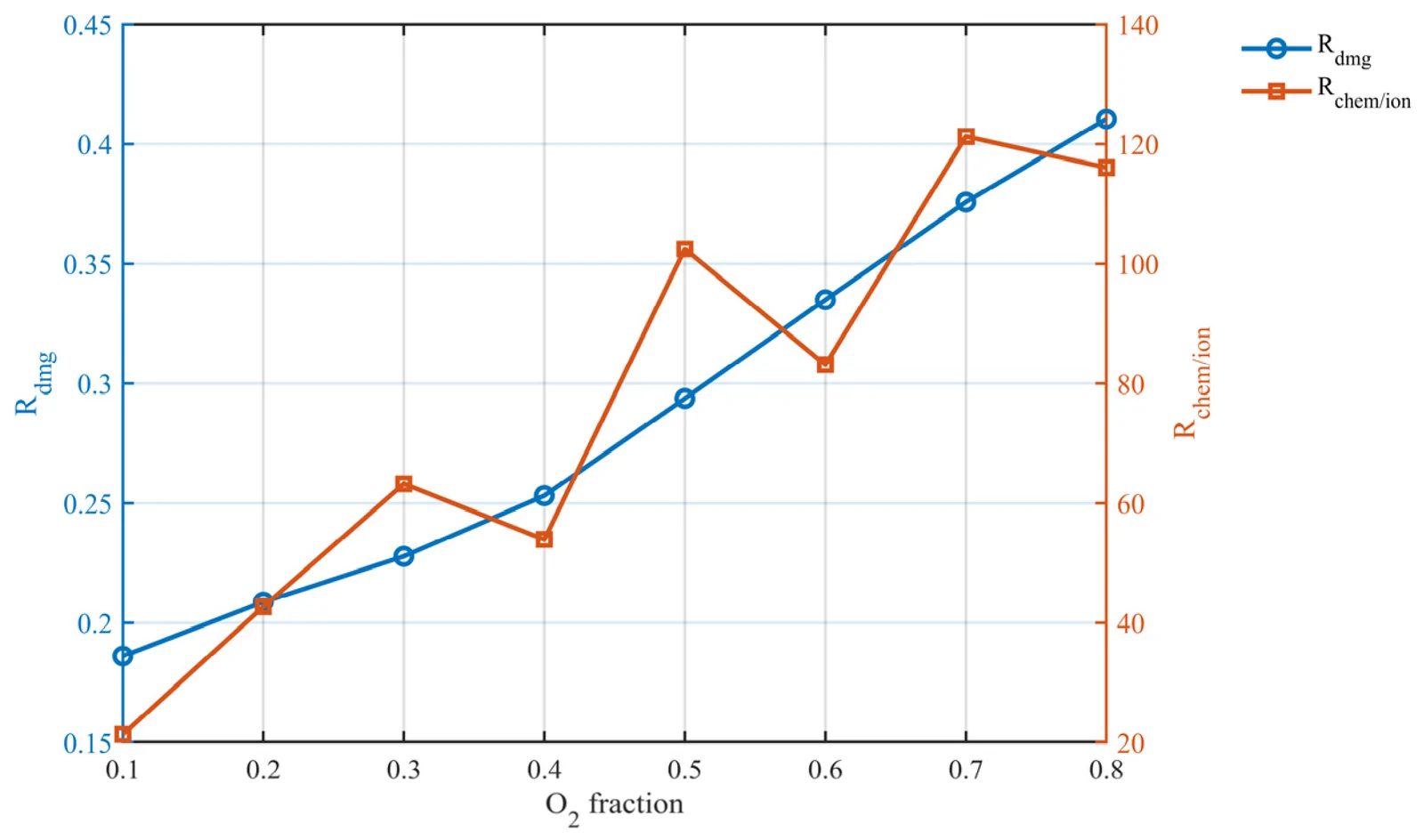
$O_{2}$-fraction dependence of the damaging ion fraction $R_{dmg}$ and the radical-to-ion flux ratio $R_{chem/ion}$. Oxygen-rich conditions enhance radical-assisted chemical contribution but also increase the above-threshold ion population, revealing a trade-off for damage-controlled $MoS_{2}$ etching.

As shown in Figure 16, $R_{dmg}$ increases monotonically from approximately 0.19 to 0.41 as the $O_{2}$ fraction rises from 0.1 to 0.8. This trend follows the increase in the maximum plasma potential shown in Figure 8: a higher $O_{2}$ fraction raises the plasma electronegativity, lowering the electron density and increasing the plasma potential. The larger sheath potential drop allows ions to gain more kinetic energy across the sheath, thereby increasing the fraction of incident ions with energies above the 50 eV threshold. This indicates an increased probability of sulfur-vacancy generation through the physical channel.

The radical-to-ion flux ratio compares the incident reactive-radical flux with the incident ion flux at the electrode,

(16) $$R_{chem/ion}=\frac{\Gamma_{O\left(3P\right)}+\Gamma_{O\left(1D\right)}}{\Gamma_{{\mathrm{Ar}}^{+}}+\Gamma_{O^{+}}+\Gamma_{O_{2}^{+}}}$$

It should be read as an incident ratio rather than as the chemical etch rate itself, since the effective probability $\gamma_{0}$ is enhanced by ion (and UV) fluxes; the ratio is built from fluxes only, and radiative (UV) fluxes are not resolved here. As shown in Figure 16, $R_{chem/ion}$ increases overall with $O_{2}$ fraction, from approximately 20 at $X_{O_{2}}$ = 0.1 to about 115–120 at $X_{O_{2}}$ = 0.7–0.8. The increase is not strictly monotonic, however, because the O-radical and total positive-ion fluxes do not scale identically with $O_{2}$ fraction; the positive-ion composition underlying the denominator is shown in Figure 6.

Overall, Figure 16 indicates a trade-off in $O_{2}$/Ar CCP processing of $MoS_{2}$. Increasing the $O_{2}$ fraction raises the relative chemical supply, but it also shifts toward higher energies and thus increases $R_{dmg}$. This suggests that an intermediate $O_{2}$ fraction may provide a more favorable low-damage processing window than either Ar-rich or strongly $O_{2}$-rich conditions.

## 4. Conclusions

In this study, a two-dimensional plasma simulation was performed to investigate the effects of the $Ar/O_{2}$ gas-mixing ratio on charged-particle distributions, plasma potential, and substrate-incident ion energy and angular distributions for low-damage MoS_2_ plasma patterning. As the $O_{2}$ fraction increased, the discharge changed from an Ar-rich electropositive plasma to an oxygen-rich electronegative plasma. The electron density decreased, whereas the $O^{-}$ density and electronegativity increased significantly, with the electronegative transition beginning at $Ar/O_{2}$ = 8:2. The dominant positive ion species also changed from $Ar^{+}$ to $O_{2}^{+}$ with increasing oxygen fraction, while $O^{+}$ remained a minor component owing to its efficient loss through charge exchange with $O_{2}$. These results indicate that the $Ar/O_{2}$ mixing ratio controls not only the plasma chemistry but also the balance between physical ion bombardment and oxygen-assisted surface modification.

The plasma potential generally increased with the $O_{2}$ fraction, leading to higher ion-energy peaks in the IEDFs; however, its nonmonotonic behavior shows that ion energy cannot be predicted by a simple linear dependence on gas composition. In addition, the two-dimensional analysis revealed that ions are incident nearly normal to the substrate in the electrode-center region, whereas a radial electric-field component near the electrode edge causes tilted and broadened ion angular distributions. Therefore, reliable low-damage MoS_2_ patterning requires simultaneous consideration of gas mixture-dependent ion species, ion energy distributions, collisional sheath transport, and edge-induced angular nonuniformity. The present simulation results provide a quantitative basis for selecting robust $Ar/O_{2}$ plasma process conditions for spatially uniform and low-damage processing of atomically thin materials.

To connect the simulated plasma environment with $MoS_{2}$ processing, the ion and oxygen-radical fluxes were further interpreted using a phenomenological two-channel surface-reaction picture. Instead of assuming uncertain surface-response coefficients, we introduced two flux-derived indicators: the damaging-ion fraction $R_{dmg}$ and the radical-to-ion flux ratio $R_{chem/ion}$. As the $O_{2}$ fraction increased, $R_{dmg}$ increased monotonically, while $R_{chem/ion}$ increased overall but not strictly monotonically. These trends reveal an intrinsic trade-off in $Ar/O_{2}$ CCP processing of $MoS_{2}$: oxygen-rich conditions enhance radical-assisted chemical modification but simultaneously increase the population of energetic ions capable of inducing sulfur vacancy-related damage. Therefore, an intermediate $O_{2}$ fraction is expected to provide a more favorable low-damage processing window than either Ar-rich or strongly oxygen-rich conditions, offering a quantitative basis for selecting robust $Ar/O_{2}$ process conditions for atomically thin materials.

The present study has several limitations. Because the $MoS_{2}$ surface was not explicitly treated as a reactive boundary, the simulation results should be regarded as plasma-side indicators rather than direct predictions of etch rate, profile evolution, oxidation, or defect density. The proposed $R_{dmg}$ and $R_{chem/ion}$ parameters also rely on flux-based interpretation without specifying uncertain surface-response coefficients, such as species-dependent sputtering yields and oxygen-assisted reaction probabilities. In addition, UV-assisted effects, detailed surface kinetics, and three-dimensional wafer-scale nonuniformity were not included. Since the present analysis was limited to a fixed geometry, pressure, RF voltage, frequency, and $Ar/O_{2}$ mixing range, the optimal intermediate-$O_{2}$ condition may shift for other reactor designs or operating conditions.

Future work should combine the current PIC-MCC plasma analysis with validated MoS_2_ surface-reaction sets and feature-profile models, including complete reactions within the kinetic framework, to quantify absolute etch rates, defect generation, and compositional modification. Such an extension will enable direct prediction of the physics of MoS_2_-specific plasma–surface interactions, building on the plasma-side insights reported here. However, reaction data have not been well-reported so far.

## Figures and Tables

**Table 1:** Simulation parameters and conditions.

Parameter	Value
Domain size (x × y)	0.064 m × 0.032 m
Grid size (Δx=Δy)	0.2 mm
Time step (Δt)	1 $\times \;10^{-11}$ s
RF voltage amplitude ($V_{0}$)	100 V
RF frequency ($f_{0}$)	50 MHz
Total feed-gas pressure	50 mTorr
$mole\;fraction\;(X_{O_{2}})$	0.1–0.8
Kinetic (PIC) species	$e^{-},Ar^{+},O_{2}^{+},O^{+},O^{-}$

## Data Availability

The original contributions presented in the study are included in the article; further inquiries can be directed to the corresponding author.

## References

[1] Altieri N.D., Chen J.K., Minardi L., Chang J.P. (2017). Plasma–surface interactions at the atomic scale for patterning metals. J. Vac. Sci. Technol. A.

[2] Profijt H.B., Potts S.E., Van de Sanden M., Kessels W. (2011). Plasma-assisted atomic layer deposition: Basics, opportunities, and challenges. J. Vac. Sci. Technol. A.

[3] Yoo J., Nam C., Bussmann E. (2024). Atomic precision processing of two-dimensional materials for next-generation microelectronics. ACS Nano.

[4] Oehrlein G.S., Brandstadter S.M., Bruce R.L., Chang J.P., DeMott J.C., Donnelly V.M., Dussart R., Fischer A., Gottscho R.A., Hamaguchi S. (2024). Future of plasma etching for microelectronics: Challenges and opportunities. J. Vac. Sci. Technol. B.

[5] Xiao D., Ruan Q., Bao D., Luo Y., Huang C., Tang S., Shen J., Cheng C., Chu P.K. (2020). Effects of Ion Energy and Density on the Plasma Etching-induced surface area, edge electrical field, and multivacancies in MoSe_2_ nanosheets for enhancement of the hydrogen evolution reaction. Small.

[6] Sovizi S., Angizi S., Ahmad Alem S.A., Goodarzi R., Taji Boyuk M.R.R., Ghanbari H., Szoszkiewicz R., Simchi A., Kruse P. (2023). Plasma processing and treatment of 2D transition metal dichalcogenides: Tuning properties and defect engineering. Chem. Rev..

[7] Liu Y., Nan H., Wu X., Pan W., Wang W., Bai J., Zhao W., Sun L., Wang X., Ni Z. (2013). Layer-by-layer thinning of MoS_2_ by plasma. ACS Nano.

[8] Zhu H., Qin X., Cheng L., Azcatl A., Kim J., Wallace R.M. (2016). Remote plasma oxidation and atomic layer etching of MoS_2_. ACS Appl. Mater. Interfaces.

[9] Lee B.J., Lee B.J., Efremov A., Yang J., Kwon K. (2016). Etching characteristics and mechanisms of MoS_2_ 2D Crystals in O_2_/Ar inductively coupled plasma. J. Nanosci. Nanotechnol..

[10] Jadwiszczak J., O’Callaghan C., Zhou Y., Fox D.S., Weitz E., Keane D., Cullen C.P., O’Reilly I., Downing C., Shmeliov A. (2018). Oxide-mediated recovery of field-effect mobility in plasma-treated MoS_2_. Sci. Adv..

[11] Kim J., Lee M., Lee W., Lee M., Kang C., Jung D., Son H., Kim E., Chae S., Kim J. (2026). Dry-transferred MoS_2_ films on PET with plasma patterning for full-bridge strain-gauge sensors. Sensors.

[12] Zhao Y., Zhou Y., Ma X., Cao L., Zheng F., Xin Y. (2019). Axial diagnosis of electron and negative ion behaviors in capacitively coupled O_2_-containing Ar plasma driven by 27.12 MHz. Phys. Plasmas.

[13] Gudmundsson J.T., Kawamura E., Lieberman M.A. (2013). A benchmark study of a capacitively coupled oxygen discharge of the oopd1 particle-in-cell Monte Carlo code. Plasma Sources Sci. Technol..

[14] Babaeva N.Y., Lee J.K., Shon J.W., Hudson E.A. (2005). Oxygen ion energy distribution: Role of ionization, resonant, and nonresonant charge-exchange collisions. J. Vac. Sci. Technol. A.

[15] Lichtenberg A.J., Vahedi V., Lieberman M.A., Rognlien T. (1994). Modeling electronegative plasma discharges. J. Appl. Phys..

[16] Dorranian D., Alizadeh M. (2014). Effect of negative oxygen ions on the characteristics of plasma in a cylindrical DC discharge. J. Theor. Appl. Phys..

[17] Lee S.H., Iza F., Lee J.K. (2006). Particle-in-cell Monte Carlo and fluid simulations of argon-oxygen plasma: Comparisons with experiments and validations. Phys. Plasmas.

[18] Vass M., Wilczek S., Lafleur T., Brinkmann R.P., Donkó Z., Schulze J. (2020). Electron power absorption in low pressure capacitively coupled electronegative oxygen radio frequency plasmas. Plasma Sources Sci. Technol..

[19] Kim C.H., Kim H., Park G., Shin J.H., Lee H.J. (2022). Two-dimensional analysis for the transition from nonlocal to local electron kinetics and its effect on the spatial uniformity of a capacitively coupled plasma. Plasma Process. Polym..

[20] Kim H.H., Shin J.H., Lee H.J. (2023). Control of the ion flux and energy distribution of dual-frequency capacitive RF plasmas by the variation of the driving voltages. J. Vac. Sci. Technol. A.

[21] Yu L., Tan J., Ma H., Liu B., Kang F., Lv R. (2025). Large-area monolayer p-Type semiconductor films toward high-performance electrical device arrays. Adv. Mater..

[22] Xue G., Qin B., Ma C., Yin P., Liu C., Liu K. (2024). Large-area epitaxial growth of transition metal dichalcogenides. Chem. Rev..

[23] Seong I., Lee J., Kim S., Lee Y., Cho C., Lee J., Jeong W., You Y., You S. (2022). Characterization of an etch profile at a wafer edge in capacitively coupled plasma. Nanomaterials.

[24] Chowdhury I., Ali M.Y., Howlader M.M.R. (2025). Advances in etching of 2D nanomaterials: Research challenges and advanced devices. Prog. Eng. Sci..

[25] Chamlagain B., Khondaker S.I. (2020). Electrical properties tunability of large area MoS_2_ thin films by oxygen plasma treatment. Appl. Phys. Lett..

[26] Islam M.R., Kang N., Bhanu U., Paudel H.P., Erementchouk M., Tetard L., Leuenberger M.N., Khondaker S.I. (2014). Tuning the electrical property via defect engineering of single layer MoS_2_ by oxygen plasma. Nanoscale.

[27] Kang N., Paudel H.P., Leuenberger M.N., Tetard L., Khondaker S.I. (2014). Photoluminescence quenching in single-layer MoS_2_ via oxygen plasma treatment. J. Phys. Chem. C.

[28] Hur M.Y., Kim J.S., Song I.C., Verboncoeur J.P., Lee H.J. (2019). Model description of a two-dimensional electrostatic particle-in-cell simulation parallelized with a graphics processing unit for plasma discharges. Plasma Res. Express.

[29] Kim J.S., Hur M.Y., Kim C.H., Kim H.J., Lee H.J. (2018). Advanced PIC-MCC simulation for the investigation of step-ionization effect in intermediate-pressure capacitively coupled plasmas. J. Phys. D Appl. Phys..

[30] Lee C., Lieberman M.A. (1995). Global model of Ar, O_2_, Cl_2_, and Ar/O_2_ high-density plasma discharges. J. Vac. Sci. Technol. A.

[31] Kastania A.S., Mouchtouris S., Stai E., Zeniou A., Kokkoris G., Constantoudis V., Tsavalas P., Mergia K., Gogolides E., Tserepi A. (2025). Plasma-induced maskless formation of quasi-periodic nanoripples on polymeric substrates. Adv. Eng. Mater..

[32] Kawamura E., Vahedi V., Lieberman M.A., Birdsall C.K. (1999). Ion energy distributions in rf sheaths; review, analysis and simulation. Plasma Sources Sci. Technol..

[33] Lu W., Birmingham B., Zhang Z. (2020). Defect engineering on MoS_2_ surface with argon ion bombardments and thermal annealing. Appl. Surf. Sci..

